# Relocation of p25α/tubulin polymerization promoting protein from the nucleus to the perinuclear cytoplasm in the oligodendroglia of sporadic and *COQ2* mutant multiple system atrophy

**DOI:** 10.1186/s40478-014-0136-4

**Published:** 2014-09-11

**Authors:** Kiyobumi Ota, Masato Obayashi, Kokoro Ozaki, Shizuko Ichinose, Akiyoshi Kakita, Mari Tada, Hitoshi Takahashi, Noboru Ando, Yoshinobu Eishi, Hidehiro Mizusawa, Kinya Ishikawa

**Affiliations:** Department of Neurology and Neurological Science, Graduate School, Tokyo Medical and Dental University, 1-5-45, Yushima, Bunkyo-ku, Tokyo 113-8510 Japan; Research Center for Medical and Dental Sciences, Tokyo Medical and Dental University, Yushima, Bunkyo-ku, Tokyo Japan; Department of Pathology, Brain Research Institute, University of Niigata, Asahimachi, Chuo-ku, Niigata Japan; Department of Pathology, Graduate School, Tokyo Medical and Dental University, Yushima, Bunkyo-ku, Tokyo Japan

**Keywords:** α-synuclein, *COQ2*, Mitochondrial fission, Multiple system atrophy, Nucleus, p25α/Tubulin polymerization promoting protein (TPPP)

## Abstract

p25α/tubulin polymerization promoting protein (TPPP) is an oligodendroglial protein that plays crucial roles including myelination, and the stabilization of microtubules. In multiple system atrophy (MSA), TPPP is suggested to relocate from the myelin sheath to the oligodendroglial cell body, before the formation of glial cytoplasmic inclusions (GCIs), the pathologic hallmark of MSA. However, much is left unknown about the re-distribution of TPPP in MSA. We generated new antibodies against the N- and C-terminus of TPPP, and analyzed control and MSA brains, including the brain of a familial MSA patient carrying homozygous mutations in the coenzyme Q2 gene (*COQ2*). In control brain tissues, TPPP was localized not only in the cytoplasmic component of the oligodendroglia including perinuclear cytoplasm and peripheral processes in the white matter, but also in the nucleus of a fraction (62.4%) of oligodendroglial cells. Immunoelectron microscopic analysis showed TPPP in the nucleus and mitochondrial membrane of normal oligodendroglia, while western blot also supported its nuclear and mitochondrial existence. In MSA, the prevalence of nuclear TPPP was 48.6% in the oligodendroglia lacking GCIs, whereas it was further decreased to 19.6% in the oligodendroglia with phosphorylated α-synuclein (pα-syn)-positive GCIs, both showing a significant decrease compared to controls (62.4%). In contrast, TPPP accumulated in the perinuclear cytoplasm where mitochondrial membrane (TOM20 and cytochrome C) and fission (DRP1) proteins were often immunoreactive. We conclude that in MSA-oligodendroglia, TPPP is reduced, not only in the peripheral cytoplasm, but also in the nucleus and relocated to the perinuclear cytoplasm.

## Introduction

p25α is a 25 kilodalton (kDa) phosphorylated protein, first identified from a tau protein kinase fraction [1]. The protein was later discovered to possess tubulin binding ability, and was renamed as tubulin polymerization promoting protein (TPPP) [2]. TPPP is expressed exclusively in the oligodendroglia in the central nervous system [1,3], and plays a critical role in myelin maturation [4]. TPPP is also essential for the reorganization and stabilization of microtubules [2,5]. Another characteristic biochemical feature of TPPP is that it normally remains as an unfolded and unstructured protein without binding partners [6], whereas in the presence of proteins such as myelin basic protein (MBP) or tubulin [2,7–9], it changes its secondary structure and exerts physiological roles such as binding with tubulin to stabilize microtubules [2,5]. TPPP is also a guanosine triphosphate (GTP)-binding protein, which hydrolyzes GTP to produce guanosine di-phosphate (GDP), and participates in multiple physiological functions [9].

In adult human brains, TPPP is considered to be expressed in the oligodendroglial cytoplasm including their terminal processes, where it plays a role in forming mature myelin by binding with MBP [8]. TPPP has also been studied in a number of human diseases. In multiple sclerosis (MS), demyelinated lesions show loss of TPPP-positive oligodendroglial cells, whereas re-myelinating areas show TPPP-upregulation in the oligodendroglial cytoplasm, subsequently followed by TPPP expression in the myelin sheath [10]. TPPP is known to accumulate in the oligogodendroglia of patients with various neurodegenerative disorders including Parkinson’s disease [11]. Multiple system atrophy (MSA) is a common neurodegenerative disorder showing parkinsonian features, cerebellar ataxia, and autonomic failure, and has been suggested to be a primary oligodendrogliopathy [12]. The neuropathologic hallmark of MSA is the formation of argyrophilic glial cytoplasmic inclusions (GCIs) in oligodendroglia [13,14], consisting of α-synuclein (α-syn) [15–19]. Previous pathological studies on human MSA brains showed that TPPP alters its distribution from the myelin sheath to the cell soma where it colocalizes with α-syn-containing GCIs [8,12].

However, much remains unknown regarding the expression of TPPP in human brains. The normal distribution of TPPP varies among previous reports: the majority of reports have emphasized that TPPP is expressed in the oligodendroglial cytoplasm and all of the processes [7,8], whereas two studies reported that TPPP is localized in the cytoplasm and nucleus [10,20]. It is not certain if this inconsistency is due to differences in the epitope locations within TPPP of the antibodies utilized. Considering that TPPP may exert various physiological functions through its GTPase activity [9,21], it is particularly important to confirm the location where TPPP is normally expressed, and how it is altered in each disease.

We therefore aimed to clarify the distribution of TPPP in normal and MSA brains. For this purpose, we generated new antibodies against the amino (N)- and carboxyl (C)-termini of TPPP, and examined human brain tissues including 11 MSA individuals. We not only confirmed that TPPP colocalizes with the myelin marker MBP, but we also found that TPPP is expressed in more than 60% of normal, mature oligodendroglial nuclei. Immunoelctron microscopy as well as western blotting also showed that TPPP is located in normal oligodendroglial mitochondria. In MSA, TPPP was lost not only from the oligodendroglial peripheral processes including myelin sheaths, but also from the oligodendroglial nuclei, and accumulated in its cell body where increased immunoreactivity was also seen for two mitochondrial membrane proteins (translocase of the mitochondrial outer membrane 20 [TOM20] and cytochrome C), and dynamin-related protein 1 (DRP1) that mediates mitochondrial fission. We investigated the redistribution of TPPP in amyotrophic lateral sclerosis (ALS), MS, and oligodendroglioma, and found that it was specific to MSA. We propose the amount of nuclear TPPP is reduced in MSA and this may perturb normal, yet unknown nuclear functions

## Materials and methods

### Subjects

Autopsies were performed with written consent from the families, and the brain and spinal cord of the patients were removed and immediately frozen at −80°C.

The study was approved by the Institutional Review Boards of Ethics of Tokyo Medical and Dental University and University of Niigata, and also conformed to the tenets of the Declaration of Helsinki. All samples were obtained either from the Department of Human Pathology, Tokyo Medical and Dental University, Tokyo, Japan, or from the Brain Research Institute, University of Niigata, Niigata, Japan.

We studied patients with sporadic MSA (Cases 1–10, 12–14), familial MSA (Case 11), who was already reported as homozygously carrying the M128V-V393A *COQ2* mutation [22,23], normal controls who died from non-neurological conditions (Cases 15–26), and disease controls including ALS (Cases 27 and 28), oligodendroglioma (Cases 29 and 30), MS (Cases 31–34) and others (Cases 35–37) (Table 1). The Case 11 as well as her/his sibling were both clinically diagnosed as MSA-P, pathologically confirmed as MSA (22), and whole genome sequence analysis on them lead to an identification of *COQ2* mutations in MSA (23).One of these two sibs, the only available sample upon investigation, were confirmed to show a significantly reduced intracellular coenzyme Q10 level in her/his brain tissue, suggesting a functional consequence of this mutation (23).

**Table 1:** **The individuals investigated in the present study**

**Case Number**	**Diagnosis**	**Age at death (years)**	**Gender**	**Disease duration**
1	MSA-C	64	M	7 y
2	MSA-C	65	F	5 y
3	MSA-C	69	M	2 y
4	MSA-C	70	M	11 y
5	MSA-C	65	M	4 y
6	MSA-C	70	F	2 y
7	MSA-C	82	M	6 y
8	MSA-C	71	M	8 y
9	MSA-C	60	M	9.5 y
10	MSA-C	71	M	11y
11	MSA-P (Familial MSA)	73	F	5 y
12	MSA-C	62	F	8 y
13	MSA-P	66	M	4 y
14	MSA-C	80	F	3 y
15	lung cancer	68	M	-
16	hemophagocytosis	67	M	-
17	polyneuropathy	64	F	-
18	gastrointestinal bleeding	75	M	-
19	abdominal hemorrhage	80	M	-
20	adult T-cell leukemia	55	F	-
21	pneumonia	61	F	-
22	acute abdomen	51	M	-
23	myasthenia gravis	82	F	-
24	Foix-Alajouanine syndrome	79	F	-
25	pneumonia	88	M	-
26	polyneuropathy	87	F	-
27	ALS	61	M	1 y
28	ALS	59	M	3 y
29	oligodendroglioma	28	F	1 m
30	oligodendroglioma	62	F	16 y
31	MS	61	F	12 y
32	MS	54	M	10 y
33	MS	31	M	7 y
34	MS	80	M	13 y
35	myotonic dystrophy	78	F	23 y
36	stroke	70	M	-
37	vitamin deficiency	31	M	7 y

M: male; F female; y: years; m: month.

### Antibodies

The anti-TPPP rabbit polyclonal antibody named TPPP-C-psB was raised by immunizing a rabbit with a synthetic peptide corresponding to the human C-terminal amino acid residues 204–219 (amino acid sequence: GYKHAGTYDQKVQGGK). The peptide was coupled to keyhole limpet haemocyanin before immunization. The anti-serum was purified on columns containing the synthetic peptide.

To generate the anti-TPPP rat monoclonal antibody (TPPP-N-mab#2A8G3), rats were immunized by subcutaneously injecting synthetic peptides corresponding to the N-terminal amino acid residues 1–39 of human TPPP (amino acid sequence: MADKAKPAKAANRTPPKSPGDPSKDRAAKRLSLESEGAG). The spleen cells of immunized rats were fused with myeloma cells, creating hybridoma cells. Clones that produced a specific anti-TPPP monoclonal antibody were identified by the enzyme linked immunosorbent assay and western blotting, and were further screened by immunohistochemistry with human brain sections, to confirm that the antibodies recognize TPPP. Other antibodies used in this study are summarized in Table 2.

**Table 2:** **Primary antibodies used in the present study**

**Antibody**	**Host**	**Dilution**	**Source**
TPPP (C-ps B)	rabbit	1:500 (IHC, IF)	our laboratory
		1:20,000 (WB)	
		1:20 (IEM)	
TPPP (N-mab#2A8G3)	rat	1:50 (IHC, IF)	our laboratory
		1:2000 (WB)	
		1:20 (IEM)	
GFAP	mouse	1:500 (IF)	Sigma
IBA1	rabbit	1:500 (IF)	Proteintech
MBP	rabbit	1:500 (IF)	DAKO
OLIG2	rabbit	1μg/ml (IF)	IBL
actin	rabbit	1:20,000 (WB)	Sigma
lamin B1	rabbit	1:500 (IF)	Santa Cruz
β-tubulin	mouse	1:5000 (WB)	BD Pharmingen
TOM20	rabbit	1:100 (IF)	Santa Cruz
		1:3000 (WB)	
calnexin	rabbit	1:20,000	Proteintech
histone H3	rabbit	1:2000 (WB)	Cell Signaling
cytochrome C	mouse	1:200 (IF)	BD Pharmingen
DRP1	rabbit	1:200 (IHC, IF)	Abcam
MFN2	rabbit	1:100 (IHC)	Proteintech
pα-syn	mouse	1:4000 (IHC,IF)	WAKO

IHC: immnohistochemistry; IF: immunofluorescence.WB: western blotting; IEM: immunoelectron microscopy.

### Preparation of mouse tissue samples

All animal procedures were performed according to the protocol approved by the Animal Experiment Committee of Tokyo Medical and Dental University. The endogenous TPPP protein level in various mouse tissues was analyzed by immunoblotting. Twelve-month-old C57BL/6J male mice were sacrificed under deep pentobarbitone anesthesia, and transcardially perfused with phosphate buffered saline (PBS). Each organ was removed and frozen immediately at −80°C. Each tissue was homogenized with 10 volumes of Tissue Protein Extraction Reagent (Thermo Scientific, Waltham, MA, USA) with protease inhibitor cocktail (Roche Applied Science, Indianapolis, IN, USA). After centrifuging at 20,000 g for 10 minutes, supernatants were stored at −80°C until immunoblotting.

### Fractionation of human brain

To clarify the expression pattern of TPPP, subcellular fractionations were performed as previously described [24,25]. All procedures were carried out in a cold room maintained at 4°C, unless specifically described. Cerebral white matter from the frontal cortex (0.1 g) of a normal control (Case 26) was homogenized with six strokes of a Potter-Elvehjem glass/Teflon homogenizer attached to a Eurostar power-control visc (IKA, Staufen, Germany) motorized stirrer set at 900 rpm with 10 volumes of buffer A (0.32 M sucrose, 1 mM EDTA, 10 mM Tris–HCl, pH 7.4) containing protease inhibitor cocktail (Roche Applied Science). The homogenate was then centrifuged at 600 × g for 10 minutes (step 1). The pellet of step 1 should contain the crude nuclear fraction, whereas the supernatant of step 1 should contain crude mitochondrial and cytoplasmic fractions including microsomal proteins. The crude nuclear fraction was diluted with 1.5 ml of 0.25 M sucrose/TKM buffer (50 mM Tris–HCl [pH 7.5], 25 mM KCl, and 5 mM MgCl_2_), and then further diluted with 3.0 ml of 2.3 M sucrose/TKM buffer, yielding approximately 4.5 ml of a 1.6 M sucrose extract. This extract was then layered onto the 2 ml of 2.3 M sucrose/buffer TKM in an SW41 centrifuge tube (Beckman, San Diego, CA, USA). Next, 1 ml of 0.25 M sucrose/TKM buffer was added on top, and centrifuged at 12,000 g for 60 minutes. The resultant second pellet was re-suspended in 1 ml of buffer A and re-centrifuged at 12,000 g for 10 minutes. The resultant third pellet was re-suspended in buffer A and re-centrifuged at 3,000 g for 10 minutes. The resultant fourth pellet was analyzed as the nuclear fraction.

The supernatant of step 1 was centrifuged at 3,000 g for 5 minutes, and the pellet was discarded. The second supernatant was collected and centrifuged at 12,000 g for 10 minutes (step 2). The third supernatant was centrifuged at 70,000 g for 60 minutes. The fourth supernatant was taken as the cytosolic fraction and the fourth pellet was microsome fraction. The resulting pellet of step 2 was suspended in 1 ml of buffer A, and centrifuged at 3,000 g. The pellet was discarded to remove contamination of nuclei. The supernatant was centrifuged at 12,000 g for 10 minutes. The second pellet was re-suspended in 1 ml of buffer A and re-centrifuged at 12,000 g for 10 minutes. The resulting pellet was considered as the mitochondrial fraction. All fractions were stored at −80°C until analysis. When analyzing with immunoblotting to provide the evidence that nuclear fraction contains TPPP, 180 ng of cytosolic, mitochondrial and nuclear proteins are loaded into each lane. To examine if microsomal fraction contains TPPP, 1.0 μg protein obtained from the second supernatant which should contain cytosolic, mitochondrial and microsomal fractions, 500 ng protein obtained from the third supernatant which should contain cytosolic and microsomal fractions, and another 500 ng protein obtained from the fourth pellet which should be the microsomal fraction, were prepared and loaded.

### Immunoblotting

Western blotting analysis was performed as described previously [25]. Transfer and detection were carried out according to the protocol provided with the ECL Detection System (Amersham Pharmacia Biotech, Piscataway, NJ, USA). Horseradish peroxidase-conjugated anti-mouse immunoglobulin G (IgG), anti-rabbit IgG, or anti-rat IgG (diluted 1:20,000, Jackson ImmunoResearch, Baltimore, PA, USA) was used as a secondary antibody.

### Immunohistochemistry

Human brains fixed in formalin and embedded in paraffin were used. Four-micrometer-thick sections were deparaffinized with xylene, washed with distilled water, and boiled for 5 minutes twice in 10 mM citrate buffer (pH 6.5). For TPPP immunohistochemistry, sections were subsequently immersed in formic acid for 5 minutes. All sections were treated with 0.3% (v/v) hydrogen peroxide in distilled water to quench the endogenous peroxide, and then incubated with normal goat, horse or rabbit sera as appropriate, for 30 minutes. Finally, sections were incubated with primary antibodies overnight at 4°C. The primary antibodies were detected with the Vectastain ABC rabbit, mouse or rat IgG kits (Vector Laboratories, Burlingame, CA, USA), and visualized using Histofine Simple Stain DAB (Nichirei Bioscience, Tokyo, Japan) according to the manufacturer’s protocol. Between each step, sections were washed three times with PBS containing 0.1% Triton-X.

### Immunofluorescence

For immunofluorescent labeling, sections were treated similarly to the immunohistochemistry procedure. The primary antibodies were detected by a one hour incubation at room temperature using either one of the following five secondary antibodies: FITC-conjugated horse anti-mouse IgG (Vector Laboratories), Alexa 555-conjugated goat anti-mouse IgG (Invitrogen, Carlsbad, CA, USA), Alexa 488-conjugated goat anti-rat IgG(Cell Signaling Technology, Danvers, MA, USA), Alexa 555-conjugated goat anti-rabbit IgG (Invitrogen), Alexa 647-conjugated goat anti-rabbit IgG (Invitrogen) (all at 1:250 dilution). Sections were examined under a confocal laser scanning microscope (LSM 510META, Carl Zeiss, Jena, Germany).

### Immunoelectron microscopy

Four-week-old C57BL6/J male mice were deeply anesthetized, perfused with 4% paraformaldehyde, and their white matter was removed. For the immunocryo-ultramicrotomy, samples were fixed in 4% paraformaldehyde in 0.1 M PBS for 30 minutes, and then immersed in 2.3 M sucrose in 0.1 M PBS for 24 hours at 4°C. The samples were mounted on a holder, frozen quickly with liquid nitrogen, and then cut using an ultracut S microtome (Reichert, Vienna, Austria) equipped with a Freezing Cryo Sectioning (FCS) system (Reichert). Frozen ultrathin (90 nm) sections were collected on formvar-coated nickel grids and then placed on droplets of 1% BSA in 0.1 M PBS. The sections were subsequently transferred to droplets of TPPP-C-psB (diluted in 1:20 with 1% BSA in 0.1 M PBS) for 12 hours at 4°C, washed with 0.1 M PBS, and incubated with goat anti-rabbit IgG conjugated with 10-nm gold colloidal particles (diluted 1:20 with 1% BSA in 0.1 M PBS, British Bio Cell International, Cardiff, UK) for 12 hours at 4°C. Subsequently, sections were stained with 1% uranyl acetate, washed with distilled water, and finally embedded with a mixture of 3% polyvinyl-alcohol and 0.3% uranyl acetate.

For the post-embedding method, samples were fixed in 4% paraformaldehyde in 0.1 M PBS for 30 minutes, and dehydrated and embedded in LR White resin. Ultrathin sections were prepared and mounted on nickel grids. After incubation with 10% normal goat serum for 10 minutes, sections were incubated overnight at 4°C with TPPP-N-mab#2A8G3 (diluted 1:20 with 1% BSA in 0.1 M PBS). We also created control samples by omitting only the primary antibodies and treating the rest of the procedures similarly. After washing with PBS, the sections were incubated with a mixture of goat anti-rat IgG conjugated to 15-nm gold particles (diluted 1:30 with 1% BSA in 0.1 M PBS, British Bio Cell International) for 2 hours at room temperature. The sections were then washed with water and stained with uranyl acetate. All sections were examined by transmission electron microscopy (H-7100; Hitachi High Technologies, Tokyo, Japan).

We randomly collected 62 mitochondria containing immune-gold particles, and analysed where the gold particles were located.

### Analysis of TPPP-immunoreactivity in the oligodendroglial nucleus

Presence of TPPP-immunoreactivity in the oligodendroglial nuclei could be assessed in single immunohistochemistry for TPPP. To confirm its nuclear existence, we also performed a double immunofluorescence study for TPPP using TPPP-N-mab#2A8G3 and an inner nuclear membrane marker lamin B1 (Table 2) and analyzed in three dimension.

### Analysis of the relationship between TPPP and GCI in MSA-oligodendroglia

The cerebral cortex and nearby white matter of the precentral gyrus, temporal and occipital lobes, putamen, cerebellum and pons were studied in selected cases. Next, the precise sequential relationship between TPPP re-localization and the emergence of α-syn-positive structures in MSA-oligodendroglia were studied in 20 individuals consisting of 10 patients with MSA (Cases 1–10) and 10 normal controls (Cases 15–24) (Table 2). Sections were subjected to triple-labeling immunofluorescence using TPPP-N-mab#2A8G3, a commonly used mouse monoclonal anti-phosphorylated α-synuclein (pα-syn) antibody (Wako, Tokyo, Japan, Table 2), and Hoechst for nuclear staining. Then, the sections were analyzed by a confocal laser scanning microscope (Carl Zeiss) with merged digital images.

We classified the oligodendroglia into six types based on the previous literatures (8, 27) and the combination of labeling patterns of TPPP and the presence or absence of pα-syn-positive GCI. We first classified the MSA-oligodendroglia into two groups according to their nuclear TPPP: those with nuclear TPPP immunoreactivity within the nucleus showing co-localization of Hoechst and TPPP (types 1, 3 or 5) and those without (types 2, 4 or 6). The oligodendroglia with both nuclear and cytoplasmic TPPP-immunoreactivities were classified as nuclear type, since the TPPP is basically a cytoplasmic protein. The oligodendroglia with pα-syn-positive GCIs was further classified into four types (types 3–6) according to the location of TPPP immunoreactivity; type 3: oligodendroglia with both nuclear and cytoplasmic TPPP; type 4: oligodendroglia with exclusively cytoplasmic TPPP; type 5: oligodendroglia with exclusively nuclear TPPP (i.e., there is no co-localization of TPPP with GCI); type 6: oligodendroglia without any TPPP-immunoreactivity, but solely with pα-syn-positive GCIs.

We randomly took photographs from 20 fields, and each oligodendroglia in these fields was classified. For type 1 and 2 oligodendrocytes, 100–150 cells were classified in each MSA and control case. For type 3–6 cells, 100–150 GCI-positive oligodendrocytes were counted randomly in each MSA case, and classified into types 3–6. As we focused on the prevalence of nuclear TPPP, we calculated the frequency of oligodendroglia that have TPPP in their nuclei in four distinct classes of oligodendroglia: 1) oligodendroglia without pα-syn-immunoreactivity (i.e., type 1/types 1 + 2); 2) all oligodendroglia with GCIs (i.e., types 3 + 5/types 3 + 4 + 5 + 6 in MSA); 3) oligodendroglia having TPPP-positive GCIs (i.e., type 3/types 3 + 4); 4) oligodendroglia having TPPP-negative GCIs (type 5/types 5 + 6). Finally, the frequencies of nuclear TPPP were compared using the Mann–Whitney test in 1) type 1/types 1 + 2 in MSA vs normal controls, 2) type 1/types 1 + 2 vs types 3 + 5/types 3 + 4 + 5 + 6 in MSA, and 3) type 3/types 3 + 4 vs type 5/types 5 + 6 in MSA. *P* values < 0.05 were considered to indicate a statistically significant difference between groups.

### Computer prediction of the classical nuclear localization signal in human TPPP

Prediction of the classical nuclear localization signal specific to the importin α/β pathway in budding yeast was performed by inputting the full-length TPPP amino acid sequence into cNLS Mapper (http://nls-mapper.iab.keio.ac.jp). With this prediction system, an NLS with a score of 8, 9, or 10, suggests that the protein is exclusively localized to the nucleus, that with a score of 7 or 8 partially localized to the nucleus, that with a score of 3, 4, or 5 localized to both the nucleus and the cytoplasm, and that with a score of 1 or 2 localized to the cytoplasm. When we tested a canonical cytoplasmic protein beta-tubulin (GenBank: AAB59507.1), there was no score indicating no NLS in the entire amino acid sequence of the beta-tubulin. On the other hand, when we tested TAR DNA-binding protein 43 [Homo sapiens; the National Center for Biotechnology Information (NCBI) Reference Sequence: NP_031401.1], a predicted score of 5.7 was produced.

## Results

### Two novel antibodies recognize endogenous TPPP that is specifically expressed in the oligodendroglia

**Figure 1:**
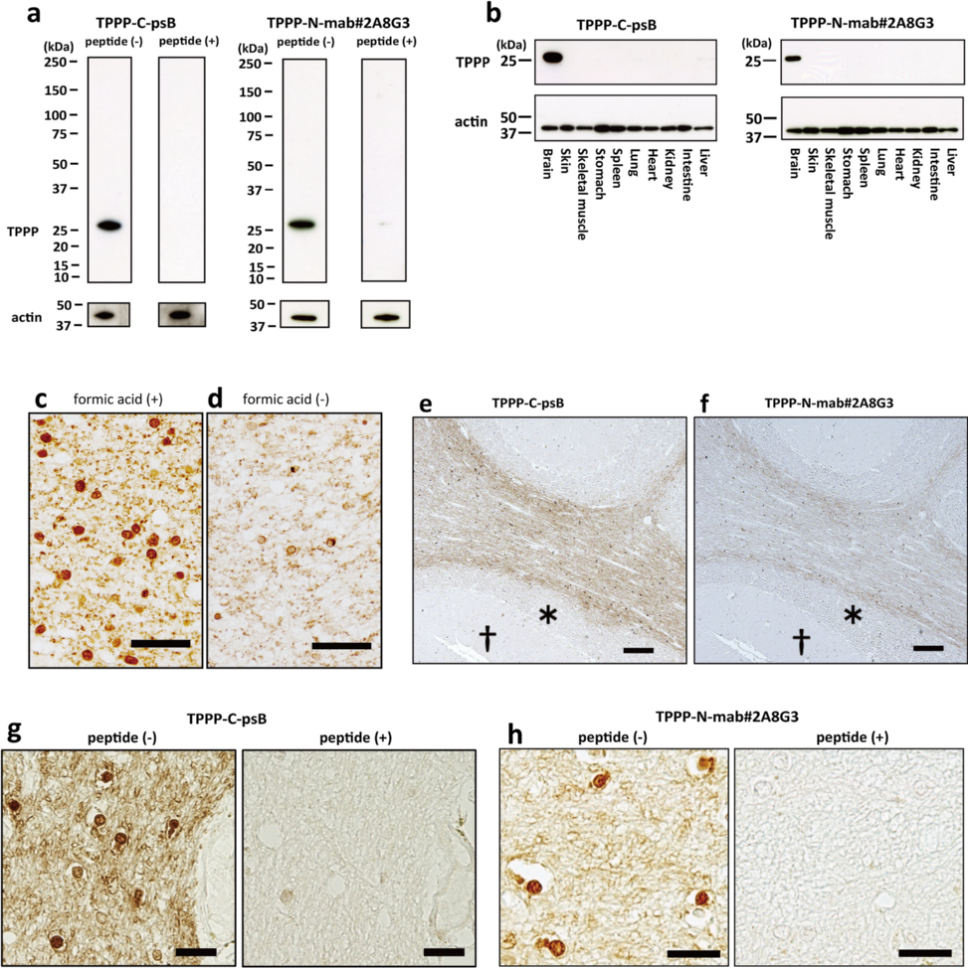
**Two novel antibodies, TPPP-C-psB and TPPP-N-mab#2A8G3 recognize endogenous TPPP that is specifically expressed in the brain. a**: TPPP-C-psB and TPPP-N-mab#2A8G3 both specifically recognize a 25 kDa protein in the mouse brain. These reactions are blocked by absorption tests (peptide (+)). **b**: Western blots of the homogenates of various organs from an adult mouse demonstrate that TPPP is a brain-specific protein. **c & d**: Some glial nuclei in the cerebellar white matter as well as perinuclear cytoplasm and neuropil show TPPP-immunoreactivity using TPPP-C-psB **(c)**, while this was not obvious in most cells when formic acid treatment was omitted **(d)** (Case 25). **e-h**: Immunohistochemistry using TPPP-C-psB **(e & g)** and TPPP-N-mab#2A8G3 **(f & h)** in normal control cerebellum (Case 25). The white matter **(e & f)** are stained intensely, whereas, granular (* in **e** and **f**) and molecular († in **e** and **f**) layers in the cortex are not. In addition to neuropil and perinuclear structures, TPPP-immunoreactivity is seen in some glial nuclei (**g & h**, peptide (−)). These reactions are also blocked by absorption tests (**g & h**, peptide (+)). Scale bar = 50 μm **(c, d)**, 200 μm **(e, f)**, 20 μm **(g, h)**.

The TPPP-C-psB and TPPP-N-mab#2A8G3 antibodies recognized endogenous mouse TPPP, and this recognition was blocked by the addition of immunogenic peptides (Figure 1a). Specificities of these two antibodies were also confirmed by testing absorptions in human frozen brain tissues (data not shown).Western blot analyses using these anti-TPPP antibodies on various mouse tissues consistently demonstrated a clear single band at 25 kDa, exclusively in the brain (Figure 1b). This brain-specific pattern of expression is compatible with a previous study using rats [3]. Upon immunohistochemical analysis, we found that the TPPP antigen was better retrieved by first boiling in citrate buffer in a microwave and then immersing it into formic acid for five minutes (Figure 1c), than by treating it with either one of these procedures only (Figure 1d). With this method, TPPP was found to be expressed most intensely in the white matter (Figure 1e & f) in low magnification. On a higher magnification, the TPPP immunoreactivity was seen not only in the oligodendroglial cell soma, processes and myelin sheath, but also in some oligodendroglial nuclei (Figure 1g & h). Of note is that the two antibodies showed the same expression patterns in immunohistochemistry, suggesting that the full-length TPPP protein is contained in the nucleus.

### TPPP is expressed in the nucleus of oligodendroglia

**Figure 2:**
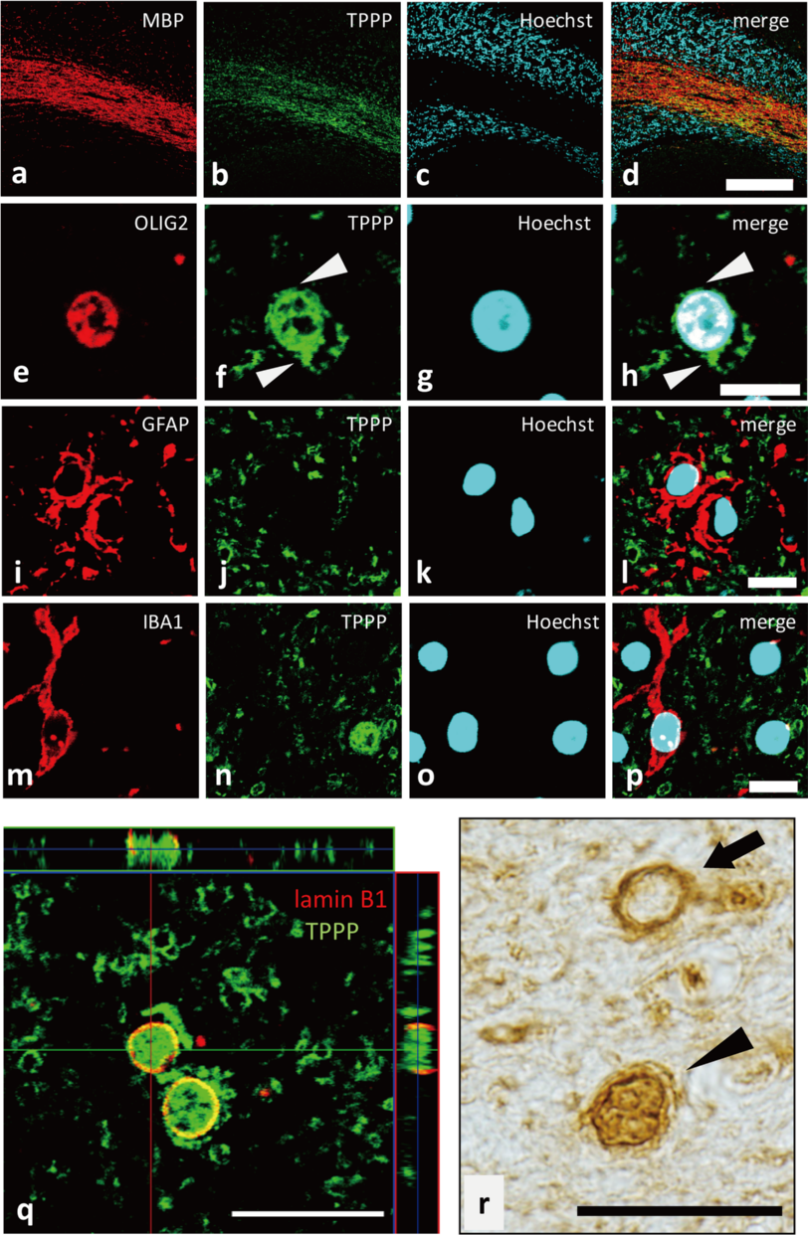
**Double-labeling immunofluorescence studies demonstrating the localization of TPPP throughout the oligodendroglia, including the nucleus.** Staining was performed for myelin basic protein (MBP) **(a, d)**, OLIG2 **(e, h)**, GFAP **(i, l)**, and IBA1 **(m, p)**, in the cerebellum of a control case (Case 25). **a-d**: Most of the MBP immunoreaction **(a)** appears co-localize with TPPP immunoreaction **(b)** in the cerebellar folial white matter. **e-h**: TPPP colocalizes with OLIG2 expressed in the oligodendroglial nucleus. Note that additional TPPP staining that does not colocalize with the OLIG2 staining (**f & h**, arrowheads), suggesting that TPPP is expressed also in the oligodendrolglial cytoplasm. **i-l**: TPPP **(j)** does not colocalize with the astroglial marker GFAP **(i)**. **m-p**: TPPP **(n)** does not colocalize with the microglial marker IBA1 **(m)**. **q**: Three-dimensional images of double-labeling immunofluorescence against TPPP and lamin B1 reveal that TPPP additionally localizes in the nucleus of oligodendroglia. **r:** Not every oligodendroglia shows nuclear TPPP staining. An arrow indicates an oligodendroglia without nuclear staining, whereas an arrowhead indicates an oligodendroglia with nuclear TPPP staining. **a-q**: TPPP-N-mab#2A8G3, **r**: TPPP-C-psB. Scale bar = 200 μm **(a-d)**, 10 μm **(e-p)**, 20 μm **(q, r)**.

Double-labeling immunofluorescence of control human cerebellum revealed that TPPP clearly merges with MBP expressed mainly in the white matter, indicating that TPPP is strongly and diffusely expressed in the oligodendroglial cell body (Figure 2a-d). OLIG2, a well-known oligodendroglial nuclear marker, colocalized with TPPP, demonstrating that some of the TPPP immunoreactivity is in the oligodendroglial nucleus (Figure 2e-h). Notably, TPPP immunoreactivity was also seen in the oligodendroglial cell soma and their processes (Figure 2f & h, arrowheads). In contrast to the clear merging of TPPP with MBP or OLIG2, TPPP did not merge with GFAP or IBA1 (Figure 2i-p), demonstrating that TPPP is not present in astroglia or microglia, respectively. We further performed double-labeling immunofluorescence against TPPP and lamin B1, a marker for the inner nuclear membrane, and confirmed that TPPP clearly localizes in the nucleoplasm using three-dimensional images (Figure 2q). These observations led us to believe that TPPP is not only present in the cytoplasm and processes including its terminal myelin sheaths, but is also present in the nucleus of oligodendroglia. However, some oligodendroglia were devoid of nuclear immunoreactivity (Figure 2r, arrow), demonstrating that oligodendroglia are heterogeneous with regard to the presence of nuclear TPPP.

### Location of TPPP investigated by immunoelectron microscopy and immunoblotting

**Figure 3:**
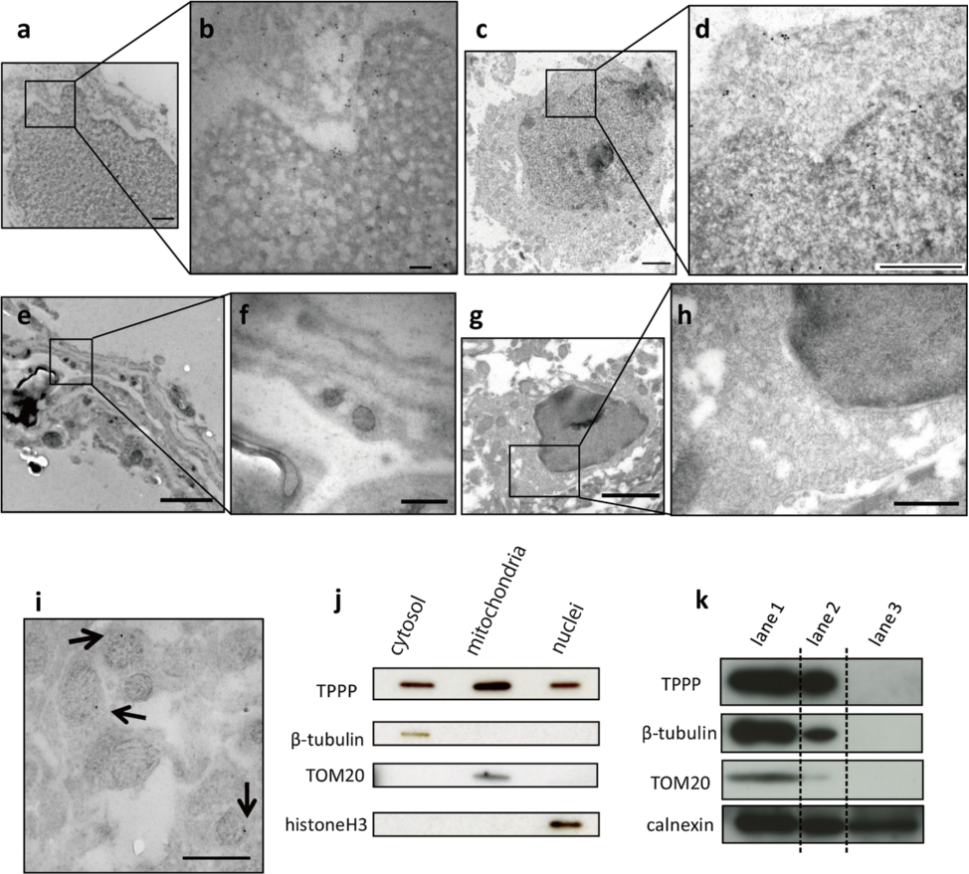
**TPPP in the nucleus and mitochondria of oligodendroglia. a & b**: Immunocryo-ultramicrotomy analysis of an adult mouse brain using TPPP-C-psB. A high magnification view of the square in panel **(a)** depicts gold particles in both the perinuclear cytoplasm and nucleus **(b)**. **c, d**: Immunoelectron microscopy of a post-embedded sample using TPPP-N-mab#2A8G3. A high magnification view of a square in the panel **(c)** demonstrates gold particles again in the cytoplasm and nucleus **(d)**. **e, f**: Gold particles are not seen on endothelial cells. **g, h**: Non-specific reactions were not seen on the samples treated with normal goat serum. **i**: Gold particles were also seen in mitochondria (arrows). **a b & j**: TPPP-C-psB, **c-f & i**: TPPP-N-mab#2A8G3. Scale bar = 500 nm **(a, d, f, h, i)**, 100 nm **(b)**, 1 μm **(c)**, 2μm **(e, g)**. **j**: Western blotting analysis of subcellular fractionation samples from human frontal lobe tissue (Case 26). TPPP-C-psB was used to detect TPPP. Anti-β-tubulin, anti-TOM20, anti-histone H3 antibodies were used as a fractionation control of cytosolic, mitochondrial and nuclear proteins, respectively. The results indicate that TPPP is present not only in the cytosolic, but also in the mitochondrial and nuclear fractions. Considering that comparable amounts of proteins are loaded, the TPPP is contained in the mitochondrial fraction **(j)**. **k**: The TPPP is not detected in the protein fraction (lane 3) which contains calnexin-positive microsomal proteins, but devoid of mitochondrial (TOM20) and cytosolic (β-tubulin) proteins.

To clarify the intracellular localization of TPPP in normal oligodendroglia, we next performed immunoelectron microscopy of 4-week-old male C57BL6/J mouse brains. Two different immunoelectron microscopic preparations (cryo-ultramicrotomy and post-embedding preparation) both consistently demonstrated gold particle deposits in the nucleus as well as the cytoplasm of oligodendroglia (Figure 3a-d). There were no particles in endothelial cells (Figure 3e & f) or in control samples that were similarly treated with normal goat serum instead of the ant-TPPP antibody (Figure 3g & h). We also noted that gold particles were localized to mitochondria. When we randomly collected 62 mitochondria containing immune-gold particles, 96 out of a total of 118 particles (81.4%) were located in the outer mitochondrial membrane (Figure 3i, arrows). This suggests that TPPP exists at least in the outer mitochondrial membrane.

We also separated the control brain tissue (frontal white matter of Case 26) into nuclear, cytosolic, microsomal and mitochondrial fractions, and analyzed them by western blotting. TPPP was detected not only in the cytosolic fraction, but also in the nuclear and mitochondrial fractions (Figure 3j). On the other hand, TPPP was not detected in the microsomal fraction (Figure 3k).

### TPPP contains nuclear localization signals

Considering the molecular size of TPPP (25 kDa), it is possible that TPPP passively diffuses into the nucleus instead of being actively transported. To gain further information, we analyzed the amino acid sequence of TPPP using cNLS Mapper (http://nls-mapper.iab.keio.ac.jp). The software predicted three potential bipartite nuclear localization signals (NLSs) in human TPPP: 1) amino acids 6–34: KPAKAANRTPPKSPGDPSKDRAAKRLSLE, with a score of 4.1; 2) amino acids 52–82: EEAFRRFAVHGDARATGREMHGKNWSKLCKD, with a score of 5.3; 3) amino acids 100–130: FSKIKGKSCRTITFEQFQEALEELAKKRFKD, with a score of 4.5. The scores ranging from 4.1 to 5.3 suggest that TPPP shuttles between the nucleus and cytoplasm by the importin α/β pathway [26].

### TPPP levels are reduced in the nucleus of MSA-oligodendroglia

**Figure 4:**
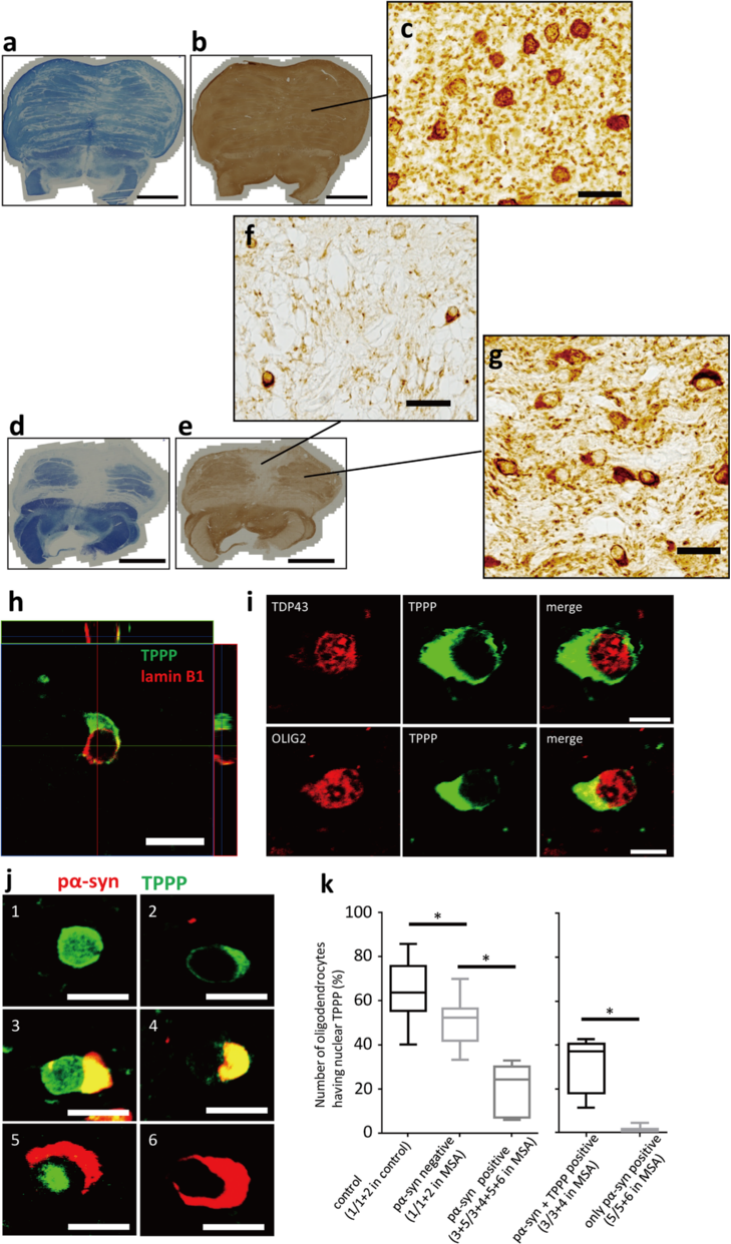
**Nuclear TPPP staining is reduced in MSA oligodendroglia. a-c**: Klȕver-Barrera staining **(a)** and an immunohistochemistry for TPPP (TPPP-C-psB) in normal control pons (Case 19). Note that myelin structures mostly show TPPP-immunoreaction, though this correlation is not always true as in the medial lemniscus and superior cerebellar peduncle. A higher magnification of **(b)** show TPPP-immunoreactivity in neuropil and in nuclei **(c)**. **d-g**: In MSA-pons (Case 8), loss of myelin structure in Klȕver-Barrera staining **(d)** is also observed in an immunohistochemistry for TPPP (TPPP-C-psB) **(e)**. Where the TPPP immunoreactivity is very low **(f)**, TPPP-positive cells are very few, suggesting oligodendroglial cell loss. In an area with relatively stronger TPPP-immunoreactivity **(g)**, TPPP-positive oligodendroglia are remaining. Note that MSA-oligodendroglia **(f & g)** tend to lack nuclear-TPPP immunoreactivity compared to control **(c)**. **h**: A three-dimensional image of double-labeling immunofluorescence of an MSA-cerebellum (Case 1) using TPPP-N-mab#2A8G3 and the anti-lamin B1 antibody. Note that nuclear TPPP is absent and the TPPP-immunoreactive cytoplasm is widened in this cell. **i**: Double-labeling immunofluorescence demonstrating the localization of TPPP (TPPP-N-mab#2A8G3) and TDP43, or TPPP and OLIG2 in the MSA-cerebellum (Case 1). TDP43 and OLIG2 are retained in the nucleus, whereas TPPP is absent. **j**: Classification of oligodendroglia into six types based on the localization of TPPP (TPPP-N-mab#2A8G3) and pα-syn. Hoechst staining is omitted from this figure. **k**: Box and whisker plot demonstrating the frequency of oligodendroglia with TPPP immunoreactivity in the nucleus. Compared with the normal control, nuclear TPPP is significantly reduced in pα-syn-negative MSA-oligodendroglia (type 1/types 1+2). The reduction of TPPP in the nucleus was more pronounced in MSA-oligodendroglia with GCIs (types 3+5/types 3+4+5+6). Nuclear TPPP was also reduced in oligodendroglia containing TPPP-negative GCIs (type 5/types 5+6) than in those containing TPPP-positive GCIs (type 3/types 3+4). *p<0.05, (k). Scale bar=5 mm **(a, b, d, e)**, 20 μm **(c, f, g)**, 10 μm **(h, j)**, 5 μm **(i)**.

As we found that TPPP normally localizes to the nucleus and mitochondria of oligodendroglia in both human and mouse brains, we then examined if the localization of TPPP is altered in MSA. Immunohistochemistry for TPPP in the pons of control individuals (Figure 4a-c) and MSA patients (Figure 4d-g) showed that TPPP is diminished in MSA in the neuropil (Figure 4f) and accumulated in the perinuclear cytoplasm of oligodendroglia (Figure 4f and g), corresponding to the relocalization of TPPP from the myelin sheath to the cytoplasm, as previously described [8,12]. In addition, we noticed that oligodendroglia possessing a swollen perinuclear cytoplasm with dense TPPP immunoreactivity frequently show loss of TPPP immunoreactivity in their nuclei (Figure 4f and g). Consistently, double-labeling immunofluorescence against TPPP and lamin B1 showed that TPPP was absent in the nuclei of the majority of MSA-oligodendroglia (Figure 4h). Importantly, TDP43 and OLIG2, two nuclear proteins expressed in oligodendroglia, were both retained in the nucleus (Figure 4i), suggesting that the reduction of TPPP from the nucleus of oligodendroglia is not a mere consequence of cellular demise. These findings were consistent in other brain areas showing degeneration, such as the precentral gyrus, putamen, and cerebellum (data not shown). In contrast, TPPP was retained in many oligodendroglia in the occipital cortex, where no obvious pathological changes were seen. Therefore, loss of nuclear TPPP immunoreactivity appears to correlate with the degree of oligodendroglial pathologic changes.

We chose the pontine base from all available areas to study if MSA-oligodendroglia lose their nuclear TPPP. Conforming to a classification of MSA-oligodendroglia reported by Huang and Halliday’s group [8,27] we classified MSA-oligodendroglia in the pontine base into six types (Figure 4i, refer to the material and methods), and compared them with the data of control oligodendroglia. Briefly, type 1, 3 or 5 cells have nuclear TPPP, type 2, 4 or 6 cells don’t have nuclear TPPP. Type 1 and 2 oligodendroglia are pα-syn negative cells, type 3–6 oligodendroglia are pα-syn positive cells. Type 1 and 2 MSA oligodendrocytes are those lacking pα-syn-positive GCIs, which are equivalent to the early pathological oligodendroglia in MSA as described previously [8,27]. The type 3 and 4 cells are middle stage, 5 and 6 cells are consistent with the late stage oligodendroglia in previous studies [8,27]. Although some reports define GCIs to include those with TPPP-positive cytoplasmic structures, we here defined “GCIs” as the cytoplasmic structures with pα-syn-immunoreactivity irrespective of TPPP-immunoreactivity.

We found that nuclear TPPP was significantly reduced in the MSA-oligodendroglia that did not possess obvious GCIs (i.e., type 1 & 2 oligodendroglia) (type 1/types 1 + 2 in MSA = 48.63 ± 10.37% vs type 1/types 1 + 2 in controls = 62.4 ± 13.5%, *p* = 0.0185) (Figure 4k). As type 1 and 2 oligodendroglia are considered to be in the relatively early stages of the pathogenic cascade of MSA [8,27], the significant reduction of nuclear TPPP appears to be a relatively early phenomenon that precedes GCI formation. In addition, the MSA-oligodendroglia containing a GCI (i.e., a pα-syn-positive cytoplasmic structure) had much less TPPP in the nucleus (types 3 + 5/types 3 + 4 + 5 + 6 in MSA = 19.6 ± 10.9%) than in the GCI-negative MSA-oligodendroglia (type 1/types 1 + 2 in MSA = 48.63 ± 10.37%, *p* < 0.001). Among the oligodendroglia with GCIs (types 3–6), the prevalence of nuclear TPPP was almost negligible in those without cytoplasmic TPPP (types 5 and 6; type5/types 5 + 6 in MSA = 0.69 ± 1.47%), demonstrating that type 5 (exclusively nuclear TPPP with a GCI) oligodendroglia are very rare. Overall, the present observations suggest that the level of TPPP in the nuclei of oligodendroglia is reduced at an early stage, before the obvious formation of a GCI (type 2) or swelling of the cytoplasm (type 4), and is followed by its eventual disappearance from oligodendroglia (type 6), at least in the pontine base that we analyzed. The pα-syn-positive glial nuclear inclusions (GNIs), neuronal cytoplasmic and nuclear inclusions (NCIs and NNIs) were so few that they could not be statistically analyzed. The relationship between TPPP and any of these structures will be separately investigated on different samples.

### Distribution of TPPP in other neurological diseases

**Figure 5:**
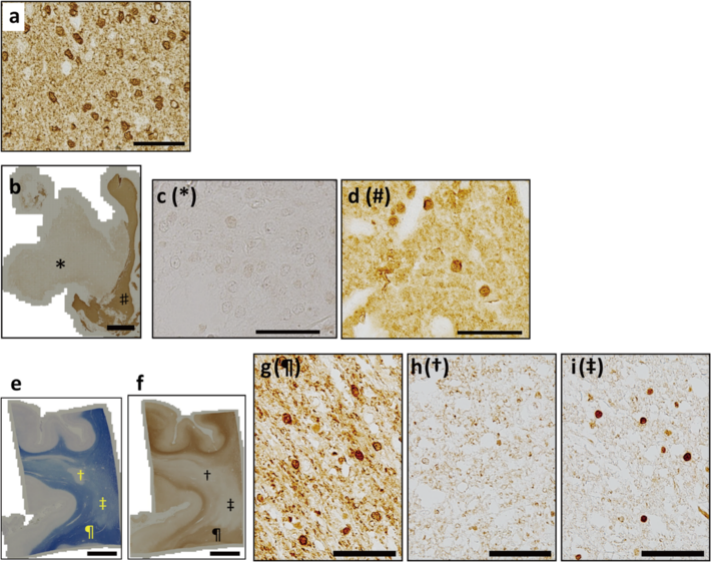
**TPPP relocalization is specific to MSA. a**: Normal staining of TPPP (TPPP-C-psB) of the frontal lobe of a patient with ALS(Case 27). **b-d**: A biopsy specimen with oligodendroglioma (Case29). Tumor mass (* in **b** and **c**) is devoid of TPPP-immunoreactivity whereas the reaction is seen in the nearby normal region (# in **b** and **d**) (antibody: TPPP-C-psB). **e-i**: A parietal lobe sample from a patient with MS (Case 31). Two corresponding panels from Klȕver-Barrera staining **(e)** and an immunohistochemistry for TPPP (TPPP-C-psB) **(f)** suggest reduced TPPP-immunoreactivity correlates with myelin loss. Relatively less affected area (¶ in **e**, **f** and **g**) shows almost normal TPPP pattern **(g)**. In an area with severe demyelination († in **e**, **f** and **h**), TPPP was nearly completely lost (h). In a different area with a milder demyelination (‡ in **e**, **f** and **i**), some oligodendroglia showed TPPP-immunoreactivity in their nuclei **(i)**. **a, b-d, f-i**: TPPP-C-psB. Scale bar = 50 μm **(a-i)**, 5 mm **(b, e, f)**.

To confirm that the relocalization of TPPP is specific to MSA, we performed immunohistochemistry for TPPP in the brains of patients with ALS, oligodendroglioma, and MS. The precentral gyrus of ALS patients exhibited no notable changes compared to normal controls (Figure 5a). In the brain tissue affected with oligodendroglioma, there was a lack of TPPP immunoreactivity (Figure 5b-d), consistent with a previous report [28].

In MS, the demyelinating areas consistently showed loss of TPPP immunoreactivity from the peripheral myelin (Figure 5e-g). However, two distinct patterns were additionally noted in terms of nuclear TPPP. One was a complete disappearance of TPPP from oligodendroglial nuclei, resulting in total loss of TPPP (Figure 5h). Such areas showed intense demyelination (Figure 5e). The other pattern was an increased TPPP staining in some oligodendroglial cell body and nuclei, with loss of TPPP from myelin (Figure 5e). Such areas showed relatively mild demyelination (Figure 5i). Although strong TPPP expression in the oligodendroglial nuclei has previously been reported, it has not received much attention [10]. It may be possible that the increase of nuclear TPPP reflects a certain defense mechanism in the oligodendroglia against cell death, or a regeneration process of oligodendroglia.

### TPPP accumulates in cytoplasm preceding pα-syn deposition in MSA-oligodendroglia along with mitochondrial proteins

Since the results of immunoelectron microscopy and western blotting after cellular fractionation both revealed that TPPP exists in mitochondria, immunofluorescence analysis for TPPP and the four mitochondrial proteins, TOM20 (a marker for the outer mitochondrial membrane), cytochrome C (a marker for the inner mitochondrial membrane), DRP1 and mitofusin2 (MFN2) [29] were performed using specific antibodies (Table 2).

**Figure 6:**
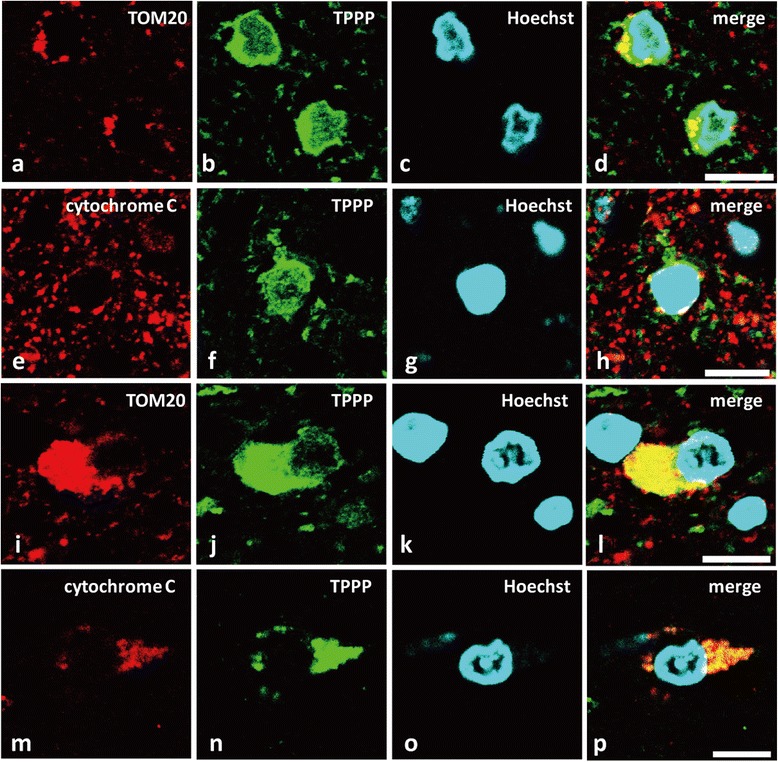
**TPPP relocates to the cytoplasm with the mitochondrial membrane markers TOM20 and cytochrome C in MSA-oligodendroglia.** Double-labeling immunofluorescence of TPPP (TPPP-N-mab#2A8G3), TOM20, and cytochrome C in a control **(a-h)** (Case 16) and an MSA patient (**i-p**, Case 1). **a-d**: In normal oligodendroglia, TOM20 **(a)**, a marker of the outer mitochondrial membrane, is detected as few small granules in the cytoplasm, whereas TPPP immunoreactivity is seen diffusely in the cytoplasm **(b)**. **e-h**: Similarly, cytochrome C **(e)**, an inner mitochondrial membrane marker, is observed as granules in normal oligodendroglia. **i-l**: In an MSA-oligodendroglia, TOM20 is accumulated in the swollen perinuclear cytoplasm **(i)**, with a similar pattern with TPPP **(l)**. **m-p**: In another MSA-oligodendroglia, cytochrome C is accumulated in the large perinuclear cytoplasm **(m)**, again in a similar pattern with TPPP **(n)**. All scale bars = 10 μm.

In normal oligodendroglia, few small TOM20-immunoreactive granules were identified in the cytoplasm (Figure 6a-d). Similarly, granular cytochrome C immunoreactivity was observed in the oligodendroglial cell body (Figure 6e-h). In contrast, both TOM20 (Figure 6i-l) and cytochrome C (Figure 6m-p) were intensely and diffusely stained in the swollen cell bodies of MSA-oligodendroglia, suggesting the accumulation of mitochondrial proteins in this pathologic condition.

**Figure 7:**
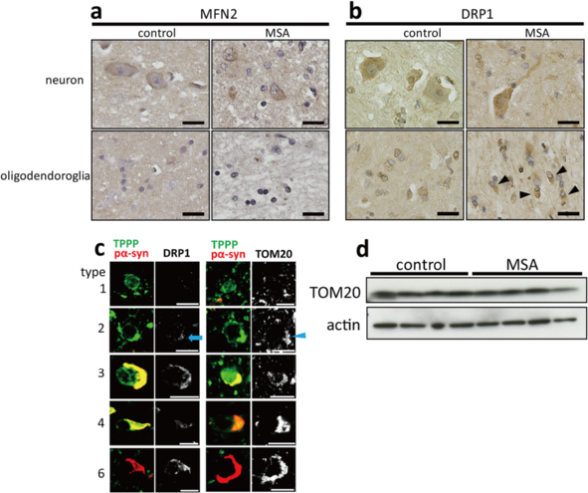
**The immunoreactivities for the mitochondrial fission marker DRP1 and the outer membrane marker TOM20 are both increased in the MSA-oligodendroglial cytoplasm before the obvious formation of GCIs. a**: MFN2-immunoreactivity is observed in the neuronal cytoplasm of both the control (Case 15) and MSA (Case 1), whereas as the immunoreaction is barely detected both in normal and MSA-oligodendroglia. **b**: DRP1 were expressed in the neuronal cytoplasm of both the control and MSA. DRP1 immunoreactivity is seen in some oligodendroglial cell bodies (arrowheads) in an MSA patient, but not obviously in control. **c**: Triple-labeling immunofluorescence for TPPP (TPPP-N-mab#2A8G3), pα-syn, and either DRP1 (left two columns) or TOM20 (right two columns). Accumulation of DRP1 and TOM20 can be seen in types 2 (a blue arrow for DRP1; a blue arrowhead for TOM20), 3, 4 and 6 oligodendrocytes. **d**: Western blotting analysis of TOM20 on subcortical white matter on precentral gyrus in control (Cases 26, 35–37) and MSA (Cases 1, 12–14) patients. There is no significant change of amount of TOM20 between MSA and controls. Scale bar = 20 μm **(a, b)**, 10 μm **(c)**.

To see if any disturbance in mitochondrial function occurs in the MSA-oligodendroglia, we finally investigated an important mitochondrial fission protein, DRP1, and a representative mitochondrial fusion mediator, MFN2 [29]. Immunoreactivities for MFN2 and DRP1 were both relatively stronger in neurons than in oligodendroglia in control tissues (Figure 7a and b, left columns). In MSA, MFN2 was also not immunoreactive (Figure 7a, right columns). Compared to such weak immunoreactivity, the DRP1-immunoreactivity was slightly increased in MSA oligodendroglia as well as in neurons (Figure 7b, right columns). In terms of the different stages of MSA-oligodendroglia, we found that DRP1-immunoreactivity was relatively stronger in GCI-containing MSA-oligodendroglia (Figure 7c; 3–6). Importantly, DRP1 immunoreactivity was observed even in the type 2 TPPP-positive (but pα-syn-negative) oligodendroglia (Figure 7c, left column, arrow). Similarly, TOM20 was also observed in type 2 MSA-oligodendroglia (Figure 7c, right column, arrowhead). These may indicate that some of the mitochondrial proteins exemplified by DRP1 and TOM20 are accumulated in the cell bodies of MSA-oligodendroglia, possibly before the obvious formation of pα-syn-positive GCIs. We tested if the mitochondrial protein TOM20 is up-regulated in the subcortical white matter of the precentral gyrus in MSA. However, we did not observe significant changes in MSA samples compared to controls. We consider that this western blot result does not preclude mitochondrial accumulation in MSA oligodendroglia observed in immunohistochemistry, as the TOM20 protein level can be influenced not only by oligodendroglia, but also by many other cells including astrocytes, microglia and neuronal processes that express TOM20.

### Pathology of familial MSA with homozygous *COQ2* mutations

**Figure 8:**
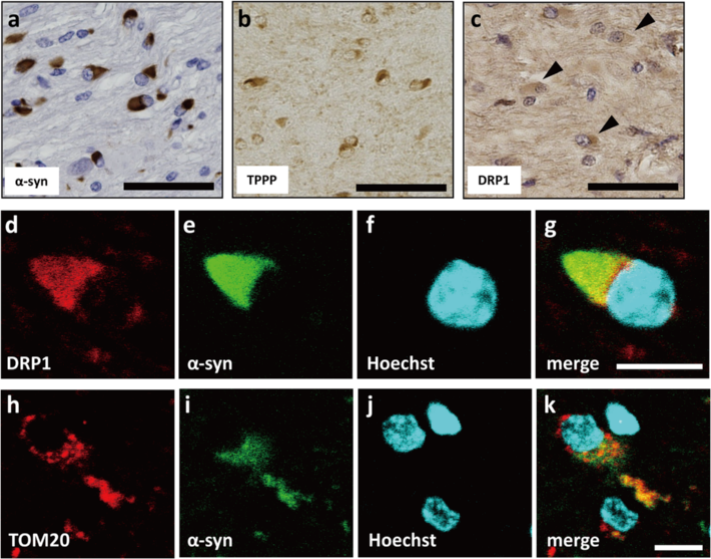
**Immunohistochemistry for TPPP, synuclein and mitochondrial proteins in a familial MSA patient with homozygous** ***COQ2*** **defective mutations.** Immunohistochemistry in the pons of Case 11 showed numerous pα-syn-positive GCIs **(a)**, the redistribution of TPPP **(b)**, and DRP1 immuno-positive GCIs (arrowheads) **(c)**. Abnormal accumulation of DRP1 **(d)** and TOM20 **(h)**, colocalizing with pα-syn **(e & i)**, can be observed in the GCI **(g & k)**. Scale bar = 50 μm **(a-c)**, 10 μm **(d-k)**.

To investigate the pathological features of MSA with defective *COQ2* variants, we studied Case 11, who had homozygous for the M128V-V393A *COQ2* mutations. Immunohistochemistry revealed pα-syn-positive GCIs (Figure 8a), abnormal TPPP distribution (Figure 8b) and DRP1-immunopositive oligodendroglia (Figure 8c). In addition, DRP1 and TOM20 co-localized with pα-syn, and accumulated in GCIs, similarly to the findings in sporadic MSA (Figure 8d-k). Although only one case was available for the present analysis, these results demonstrate that the *COQ2* mutation, leading to Coenzyme Q10 deficiency, causes TPPP relocation. Furthermore, identical TPPP redistribution in our sporadic and familial MSA samples suggests that the redistribution of TPPP and the abnormal accumulation of mitochondrial proteins are a common pathway in the MSA pathophysiology of both sporadic and familial cases.

## Discussion

By generating two antibodies against TPPP, we here confirmed that TPPP is expressed exclusively in the brain, and further that the expression is restricted to oligodendroglia, as previously reported [3]. We also found that TPPP is expressed abundantly in the cytoplasm and processes including the myelin sheath as previously described. In addition, the TPPP was seen in the nucleus of about 62% of normal oligodendroglia, when analyzed in the pontine base. Considering that oligodendoglia are a group of cells with different biochemical and metabolic properties [30], it is not surprising that TPPP is not observed in all oligodendroglial nuclei. The presence of TPPP in the nucleus was consistent: it was demonstrated by (1) immunohistochemical and immunoelectron microscopic analyses of human and mouse brain tissues, (2) double-labeling immunofluorescence analysis for TPPP and lamin B1 with three-dimensional visualization and (3) western blotting of nuclear, cytosolic and mitochondrial fractions of human brain homogenates. Whereas most previous reports emphasized that TPPP was expressed in the oligodendroglial cytoplasm and all oligodendroglial processes [7,8], two studies previously reported that TPPP is localized in the nucleus [10,20], consistent with the present data. Höftberger and colleagues previously analyzed the localization of TPPP in the human brain by immunohistochemistry and immunoelectron microscopy, and showed that TPPP was localized in oligodendroglial nuclei as well as its cytoplasm [10]. Acevedo and colleagues also found similar nuclear localization of TPPP in the non-neuronal cell lines NIH-3T3 and HeLa using immunocytochemistry [20]. As the present investigation and Höftberger’s study both utilized formic acid to retrieve hidden antigen, formic acid pretreatment may be required to detect nuclear TPPP in human formalin-fixed tissue samples. More importantly, the present observation of the nuclear localization of TPPP was confirmed with antibodies against both N- and C-termini of TPPP, suggesting that the full-length protein exists in the oligodendroglial nucleus. The molecular size of TPPP (25 kDa) suggests that this protein can passively diffuse into the nucleus from the cytoplasm through the nuclear pore [31,32]. However, our finding of three predicted bipartite nuclear localization signals in TPPP indicates that this protein has the potential to translocate into the nucleus through the importin α/β pathway, which is highly conserved in eukaryotes [26]. Increased nuclear TPPP expression in demyelinating MS plaques also suggests an active role of TPPP in the nucleus. Then, what is the role of TPPP in the nucleus? Interestingly, TPPP has the classical zinc finger domain His_2_Cys_2_ at the amino acid position 61–83 [9]. Although binding of a zinc ion (Zn^2+^) to this site has been shown to stabilize the structure of TPPP and to promote tubulin assembly in the presence of tubulin in the cytoplasm [9], its possession of a classical zinc finger motif also implicates its role as a transcription factor via binding to DNA in the nucleus. It is interesting to note that OLIG1, another oligodendroglia-specific protein known as a helix-loop-helix transcription factor in the nucleus, can translocate into the cytoplasm, and when phosphorylated, facilitates membrane expansion leading to the maturation of oligodendroglia [33]. It is thus possible that TPPP, another phosphorylated protein, has a dual role depending on its location (i.e., the nucleus or cytoplasm) within oligodendroglia.

To the best of our knowledge, the present study is the first to show that TPPP is reduced in the oligodendroglial nucleus of patients affected with MSA. The reduction of TPPP from the nucleus is not a secondary phenomenon related to global cellular dysfunction, since other authentic nuclear proteins such as TDP43 and OLIG2 were both retained in the nucleus. We also confirmed the results of previous studies describing that the immunoreactivity of TPPP was markedly decreased in the peripheral processes of MSA-oligodendroglia, whereas TPPP was increased in the swollen perinuclear cytoplasm of some MSA-oligodendroglia [8,11,12,27,34,35]. These observations indicate that TPPP not only relocates from the terminal processes that normally form the myelin sheath, but also translocates from the nucleus and focally accumulates in the swollen perinuclear cytoplasm. Song and colleagues also analyzed the pontine base, and reported that the accumulation of TPPP in the oligodendroglial perinuclear cytoplasm precedes the generation of obvious pα-syn-positive GCIs, the pathologic hallmark of MSA [8]. Our findings are consistent with this study, as TPPP was found reduced in the oligodendroglia lacking GCIs (MSA type 1/types 1 + 2 = 48.63 ± 10.37% vs type 1/types 1 + 2 in controls = 62.4 ± 13.5%; *p* = 0.0185). In addition, the prevalence of nuclear TPPP was further reduced among the MSA-oligodendroglia with pα-syn immunoreactivity (i.e., GCI-positive oligodendroglia) (types 3 + 5/types vs 3 + 4 + 5 + 6 in MSA = 19.6 ± 10.9%, *p* < 0.001). Taking the present results together with those of Song and colleagues, TPPP appears to relocate from both the terminal processes and the nucleus to the perinuclear cytoplasm in oligodendroglia prior to the formation of GCIs. Yet, it is not clear how the relocalization of TPPP associates with the aggregation of pα-syn in oligodendroglia. A previous study showed that α-syn is detected in rat primary culture oligodendroglial cells [36], suggesting that it could be endogenously upregulated under certain conditions. Alternatively, α-syn may enter oligodendroglia through dynamin-mediated endocytosis, as shown in oligodendroglial cell lines [37,38]. Once α-syn is expressed in oligodendroglia by means of yet unidentified mechanisms, it is conceivable that TPPP and α-syn bind to each other to form aggregates [34,37,38] and to promote the phosphorylation of α-syn at Serine 129, leading to microtubule retraction followed by oligodendroglial cell death [39]. If this is the case, TPPP relocalization could be an important upstream target for developing treatments against MSA. However, it should also be noted that the appearance of GCI may not always indicate a late stage event in oligodendroglia. To clarify oligodendroglial pathology, we need to investigate by different methods, such as cultured cells.

The present study also provides evidence that TPPP is a mitochondrial protein. Electron microscopic analysis combined with immunohistochemistry suggested that TPPP localizes to mitochondria, most frequently to the outer membrane. Subcellular fractionation experiments suggested that this protein localizes at least in mitochondria. Although the precise function of TPPP in mitochondria is completely unknown to date, it may be possible that TPPP plays a role as a mitochondrial motor protein, considering its binding capacity to tubulin [40]. Similar to other mitochondrial membrane proteins such as MFN1, MFN2 or DRP1 [29,41], TPPP demonstrates GTPase activity in the presence of zinc [9] and magnesium [21] ions, suggesting its multiple roles in physiological dynamics (for example, signal transduction).

We further demonstrated that TPPP is accumulated in MSA-oligodendroglial cell soma, where the mitochondrial proteins TOM20, cytochrome C, and DRP1 also showed stronger immunoreactivity compared to controls. The present observations using triple immunolabeling for TPPP, pα-syn, and either TOM20 or DRP1, indicate two potentially important aspects of MSA-oligodendroglia. One is that both DRP1 and TOM20 are accumulated in the pα-syn-positive GCI of MSA-oligodendroglia. Mitochondria dynamically control their quality through fusion and fission, creating their network in the cytoplasm [29]. When the important mitochondrial fission mediator DRP1 is over-activated, it can lead to small and discrete mitochondria (mitochondrial fragmentation), and the generation of reactive oxygen species, and the mitochondria can be subjected to mitophagy in order to destroy malfunctioning mitochondria [29,41]. In a number of neurodegenerative conditions, mitochondrial fission is increased whereas fusion is impaired, resulting in mitochondrial fragmentation [41]. Based on the present data, MSA oligodendroglia may be deviated toward in the hyper-fission state. In line with this view, parkin, a ubiquitin E3 ligase relevant to mitophagy, has been shown to be positive in some GCI-positive oligodendroglia [27]. In addition, small ubiquitin-like modifier type 1 (SUMO-1), which regulates DRP1 activity, is also positive in some GCIs [42]. Therefore, it is possible that our finding of substantial DRP1 immunoreactivity in GCI-containing oligodendroglia reflects abnormal mitochondrial fission leading to mitochondrial fragmentation and mitophagy.

The other important implication from this triple-labeling experiment is that both DRP1 and TOM20 start to appear before the obvious deposition of pα-syn in MSA-oligodendroglia. This indicates that abnormal mitochondrial accumulation, as well as fission, possibly precedes GCI formation. The Multiple System Atrophy Research Consortium reported that functionally impairing nucleotide variants in *COQ2*, which encodes the protein coenzyme Q_2_ that is essential for the biosynthesis of coenzyme Q_10_, were associated with an increased risk of MSA in multiplex families and in about 10% of sporadic MSA cases [23]. Patients with defective *COQ2* mutations were shown to have GCI-positive oligodendroglia [23], providing direct evidence for a role of mitochondrial dysfunction in MSA pathogenesis. In the present study, we were able to investigate a patient with this defective *COQ2* mutation, and found that the changes in the levels of TPPP and the mitochondrial proteins TOM20 and DRP1, as well as the reduction of nuclear TPPP, are similar to what we observed in sporadic MSA individuals.

In conclusion, we found that TPPP normally localizes abundantly in the cytoplasm, but also in the nucleus of some oligodendroglia. TPPP is localized in mitochondria as well as other cellular component such as cytosol. Based on our findings and previous descriptions [8,12], in MSA, we hypothesize that TPPP can relocates not only from the peripheral processes but also from the nucleus, and accumulates in the perinuclear cytoplasm. Deposition of pα-syn and the eventual disappearance of TPPP appear to follow such TPPP relocation, if our classification of MSA-oligodendroglia truly reflect MSA-oligodendroglial pathologic cascade. This hypothesis requires further evaluation from different aspects. For example, technical limitations, such as differences in the affinity of the various antibodies to their immunogen (i.e., anti-TPPP-antibody vs anti-α-syn-antibody) should be carefully re-considered. *In vitro* experiments such as using oligodendroglial cell line is required to make the hypothesis evident. Nevertheless, the present study suggests the need to discover the role(s) of TPPP in the nucleus and mitochondria, and how they are disturbed in MSA. Concerning the TPPP and the mitochondrial proteins accumulations in the cytoplasm of MSA-oligodendroglia, the present observation on the *COQ2*-mutant MSA case implies that the primary mitochondrial dysfunction can lead to the TPPP relocation secondarily. It is also not known how TPPP relates with these mitochondrial proteins in a molecular level. Addressing these questions will provide new information on potential roles of TPPP in both normal oligodendroglial biology and MSA pathogenesis.
